# Immediate and antecedent causes of mortality in hospitalised Indian patients with COVID-19

**DOI:** 10.6026/97320630018402

**Published:** 2022-04-30

**Authors:** BY Keerthi, K Saritha, Chirali Shah, Vimala Thomas, Vikram Cheryala

**Affiliations:** 1 Department of General Medicine, Telangana Institute of Medical Sciences and Research, Gachibowli, Hyderabad, India, 500032; 2 Department of Family Medicine, Telangana Institute of Medical Sciences and Research, Gachibowli, Hyderabad, India, 500032; 3 Department of Pulmonary Medicine, Telangana Institute of Medical Sciences and Research, Gachibowli, Hyderabad, India, 500032; 4 Department of Preventive and Social Medicine, Telangana Institute of Medical Sciences and Research, Gachibowli, Hyderabad, India, 500032; 5 Department of Family Medicine, Telangana Institute of Medical Sciences and Research, Gachibowli, Hyderabad, India, 500032

**Keywords:** COVID-19 infection, Comorbidities, immediate and antecedent cause of mortality, anaemia

## Abstract

It is of interest to assess the immediate and antecedent causes of mortality amongst adult COVID-19 infected patients with or without comorbidities admitted in an exclusive COVID-19 hospital was conducted the between August 2020 to May 2021. The immediate and antecedent causes were collected from the medical certificate of cause of death (MCCD). Remaining data was extracted from the hospital’s record. ICMR protocol was used to grade severity of illness at admission into mild, moderate and severe categories. Clinical status during hospitalisation and most recent radiographic and laboratory data were used to assess disease progression and outcome. This study includes data from 571 people, who died at our centre between August 2020 and May 2021. Patients registered without any co-morbidity were 146 with mean age of 57.53 years; (33/146) were females and (110/46) males. Hypertension (274, 47.99%) was found in a moderately large number of patients followed by diabetes (225, 39.4%) and anaemia (199, 34.6%). Increase in risk of mortality of COVID-19 was found maximum in patients with acute respiratory distress syndrome (72.33%), followed by secondary infections (6.83%). Mortality recorded in this study was mainly in males of older age (50 years and above) with at least one co-morbidity. Anaemia was also prevalent amongst these patients and considered as an independent factor for mortality. Hence, recording of comorbidities and haemoglobin levels may help as a guideline to develop risk stratification and management of patients with COVID-19 to reduce overall mortality.

## Background:

The novel corona virus (SARS-CoV-2) is an emerging disease that was first diagnosed in China and has been declared as a pandemic by the World Health Organisation on 11th March 2020. This virus belongs to the family of beta corona virus that causes severe acute respiratory syndrome [1]. SARS-CoV-2 (severe acute respiratory syndrome corona virus 2) has infected tens of millions of individuals around the world, leading to substantial mortality [2] A number of factors have been linked to an increased risk of COVID-19-related catastrophic consequences and mortality [3] With increasing age, mortality appears to soar exponentially. Higher risk is also linked to male gender, obesity, socioeconomic deprivation, and a variety of pathologies [4,5]. COVID-19 is primarily a respiratory illness. It can also cause symptoms that are not related to the lungs. Thrombotic complications, myocardial dysfunction and arrhythmia, acute coronary syndromes, acute kidney injury, gastrointestinal symptoms, hepato-cellular injury, hyperglycaemia and ketosis, neurologic illnesses, ocular symptoms, and dermatologic complications are some of the conditions that can occur [6]. However, there is scarcity of data on how the factors linked to COVID-19 mortality are associated with different comorbidities. Advanced age is also considered as a substantial risk factor of mortality from any cause. It is possible that COVID-19 infection merely increases everyone's chance of death by a constant factor, or that some things have a different impact on COVID-19 mortality. COVID-19-relatedmortality cannot be attributable to another disease (e.g., cancer) and should be counted separately from pre-existing conditions suspected of precipitating a severe course of COVID-19 [7, 8]. Day of death was identified as the time between the inception of symptoms and signs and the moment of death [9]. The vital causes of death recorded due to COVID-19 disease are acute respiratory distress syndrome, pneumonia and comorbidities. [8] In most of the cases it is likely that COVID -19 is the underlying cause of death (UCOD) which leads to ARDS resulting in mortality amongst positive patients. While recording form 4 medical certification of cause of death (MCCD), it is necessary to underline the pre -existing comorbidities that might have contributed to severity and mortality of patients [7]. Studies done on mortality with this infection suggested that; patients who died due to COVID-19 infection suffered from at least one of the comorbidities such as hypertension, diabetes, heart disease and cancer [10,11]. There is a lack of literature on studies assessing immediate and antecedent causes of mortality amongCOVID-19 infected patients, along with association of comorbidities. Therefore, it s of interest to assess immediate and antecedent cause of mortality amongst patients with COVID-19 infection admitted in an exclusive COVID-19 hospital.

## Materials and Methods:

### Study setting and design:

A retrospective, observational single-centre study was designed to assess major causes of mortality associated with COVID-19 infection. Inclusion criteria were marked as death due to COVID-19 amongst the patients with and without comorbidities. Case sheets of all the deceased COVID-19 infected patients aged 18 or more who died during hospitalisation between August 2020 till May 2021, with SARS CoV-2 confirmed by RT-PCR or rapid antigen test (RAT) were taken on record. Demographic and clinical characteristics such as age, gender, day of illness at admission, symptoms at admission, duration of hospital stay, severity at admission, underlying chronic disease histories (diabetes, hypertension, cardiovascular disease, respiratory disease and cancer) and day of death from symptom onset were extracted from the hospital's record. Since the data were collected during the peak of the pandemic, information regarding onset and duration of symptoms at time of admission was missing from a few patients (15 cases). ICMR protocol was used to grade severity of illness at admission into mild, moderate and severe categories. The days of illness was divided into early presentation which represents patients suffering from disease > 7 days, and late presentation in which onset of illness was <7 days. Clinical status during hospitalisation and most recent radiographic and laboratory data were used to assess disease progression and outcome. Study outcome was mortality, defined as the proportion of patients who died from COVID- 19 in the hospital. The immediate and antecedent causes of mortality were collected from the MCCD and confirmed after a comprehensive review of the case data. The cause of mortality was categorised as immediate, antecedent and due to pre-existing comorbidities. The ICD 10 code was used to record the various causes of death.

### Ethical approval:

The study was conducted after seeking approval from institutional scientific and research committee. Informed consent was taken at time of admission and permission was sought to access the deceased person's medical records.

### Statistical analysis:

The R software version 4.1.1 and Microsoft Excel were used to analyse the data. A frequency table was used to show categorical variables. Continuous variables were shown in Mean SD/ Median (Min, Max) format. The Chi-square test was used to determine if categorical variables are interdependent. The effect of several variables on the likelihood of developing severe COVID-19 infection is estimated using uni-variate and multivariate logistic regressions. Statistical significance is indicated by a P-value of less than or equal to 0.05.

## Results:

### Characteristics of the subjects:

The ages of patients ranged from 23 to 93, with a mean age of 57.67 ± 13.08 years. The majority of the participants were over the age of 50-59 (27.5%) followed by 60-69 (25.92%) years. The gender ratio was 1.52:1, with 60.25 % of males and 39.75 % of females. Severity of illness at the time of admission was notable, with 505 (88.44%) patients in severe condition. ICU admission was required for 443 patients (77.58%), whereas ward admission was required for 128 (22.42 %). In 94.57% of the subjects, the day of death was within 7 days. Admission was delayed (> 7 days) in 48.69% of the cases. In 54.82% of cases, the length of hospital stay was more than seven days. Information about day of death was available for only 566 patients as compared to total 571 since patients admitted in severe stages could give data on when the infection started. Only 564 subjects had detailed information regarding their symptoms on their day of admission (day of illness from the onset of symptoms) resulting in a smaller number of patients (Table 1 - see PDF).

### Immediate and antecedent causes of mortality:

Among all SARS-CoV-2 deaths, the most common immediate cause of mortality was identified as acute respiratory failure (71.8%) as shown in Figure 1a. Pathologies associated with mortality (Table 2 - see PDF) were sepsis (8.06%), cardiac failure (3.8%) as shown in Figure 1b and cardiogenic shock (3.68%) diabetic ketoacidosis (3.5%) as shown in Figure 1c(see PDF). Although a clear pattern is not observed in pathologies responsible for mortality due to COVID-19, most of the cases had at least a single comorbidity (Figure 1 - see PDF). Patients registered without any comorbidity were 146 with mean age of 57.53 years; (33/146) were females and (110/46) males. Increase in risk of mortality of COVID-19 was found maximum in patients with acute respiratory distress (72.33%), followed by secondary infections (6.83%), COVID pneumonia (6.13%) and infection (5.25%). Myocardial infarction and cardiac dysrhythmia were also found prominent in patients with COVID infection (4.55% and 2.28% respectively).

### Risk factors associated with severe infection:

Age, duration of hospital stays, symptoms at admission such as sore throat, dyspnea, myalgia, diarrhoea, headache, vomiting, and cardiovascular diseases are observed to have significant association with severe infection, according to univariate logistic regression. The risk of severe COVID-19 infection rose by 0.97 % per unit increase in age. When comparing patients with a hospital stay of ≥ 7 to those with a hospital stay of < 7, the odds of developing severe COVID increased by 0.42 %. The odds of having severe COVID rise by 0.44, 2.51, 0.53, 0.34, 0.18, 0.22, and 0.37 % for those who have a sore throat, dyspnea, myalgia, diarrhoea, headache, vomiting, and cardiovascular illness, respectively (Table 3 - see PDF). In multivariate logistic regression (Table 3 - see PDF) also similar association was observed with, age (P=0.0102), hospital stay (P=0.0010), headache (P=0.0151), vomiting (P=0.0085), total leucocyte count (P=0.0446), and neutrophil count (P=0.0305); all had a significant effect on severity. The probabilities of severe COVID-19 infection rise by 0.97, 1.22, and 0.8 % with each unit increase in age, total leucocyte count, and neutrophil count, respectively.

### Trend of age and gender of COVID-19 related mortality in first and second wave:

The first wave peaked at the end of August 2020 and was followed by a progressive decrease with very few cases admitted in hospital by mid of November and December. The number of patients who died in first wave in the hospital were 49; out of which 21 (42.86%) were ≥70 years of age (Figure 2a - see PDF), 13 (26.53%) were in between 60-69 years, 10 (20.21%) belonged to age group of 50-59 and remaining 10% aged below 50. The second wave peaked around mid March of 2021 and declined around May 2021. The number of patients deceased in the centre were around 522. Maximum i.e 143 (25.04%) deaths were observed in the age group of 50-59 years followed by 142 (24.87%)between 60-69years, 116 deaths (20.23%)above ≥ 70 years and remaining 10.2% of deceased were below 50 years. The patients who died were significantly older (70 years and above) in the first wave with a mortality rate of 42.86% and in the younger age group (18 to 50 years) mortality was around 4.08%. However, in the second wave younger patient's mortality was higher (25.04%) than the older patients. However, in both waves, the male (Figure 2b - see PDF) mortality rate was higher (63.23% and 62.87%) than females (36.73% and 37.13%). According to the data obtained, the second wave of COVID-19 affected younger patients with at least one of comorbidities than the first. However, more studies should be conducted to compare mortality related to COVID'S first and second waves. Figure 2(see PDF) below visualises the findings trend.

## Discussion:

The objective of this study was to observe the immediate and antecedent cause of mortality in patients suffering from COVID-19. In our study, an increase in mortality was reported in older age groups (59 years to 70 years); similar patterns were observed in other countries affected by COVID-19. In a hospital-based study of India on 425 patients, reported old age (65 years and above) as a confounding factor to increasing risk of death in COVID-19 patients.[4] In research from Asia, Europe, and North America, age-specific mortality rates were relatively similar. [12] A plausible reason for this age-related mortality could be chronic medical conditions and lower-level of immunity. We found an overall mortality higher in wave 2 (37.33% females and 62.87% male) than in the first wave (36.7% females and 63% males). Patients of age 50 to 70 years recorded an increase in mortality in the 2nd wave (25.04%) compared to the 1st wave (20.4%), with a higher number of mortalities in male patients than females in both the waves. In other age groups also, mortality was observed higher in the second wave. Similarly, in a retrospective study from North India, that included 10 tertiary care units, concluded an increase in mortality in the patients in wave 2 as compared to wave.[13] However, a study done by Jain et al.., showed no significant increase in mortality rate amongst both the waves. [14] Interestingly the result of study in Spain showed a decrease in mortality rates by 13.2% in the second wave as compared to (24%) in the first wave. [15] The increase in mortality rate during the second wave was reported by many countries, despite improved treatment protocol. This could be attributed to the lack of COVID- 19 appropriate behaviour (mask use and maintaining social distancing) amongst the people after vanishing of 1st wave and due to the urge of returning to normalcy. However, there is a dearth of articles comparing mortality rate amongst first and second waves. Hence, more studies should be done in order to provide evidence-based results.

## Symptoms at time of admission:

COVID-19 is difficult to identify since its symptoms are often mistaken with those of other chronic disorders. As a result, analysing and mining relationships between symptoms could aid in diagnosis. SARSCoV-2 can produce severe respiratory symptoms, such as fever and dry cough, similar to middle-east respiratory syndrome coronavirus (MERSCoV), which also causes similar respiratory infections. Some people experience digestive symptoms first, rather than respiratory symptoms, such as decreased appetite, lethargy, nausea, vomiting, and diarrhoea. Diarrhoea is a zoonotic symptom that 20% to 25% of MERSCoV or SARS-CoV affected people [16]. Symptoms of the neurological system, such as headaches, and cardiovascular symptoms, such as palpitation and chest discomfort, were also common in many patients. All these symptoms were experienced by the subjects in this study. It was discovered that gender differences in symptoms were statistically significant, albeit of a smaller extent. In our study, fever, cough, and shortness of breath were more common in men than in women; however, all other symptoms were equally or more common in female patients. In a group of non-hospitalized COVID-19 patients in Poland, there were more variations in symptoms with lack of appetite (55% of women, 36 % of males) and loss of taste (53% women, 40 % men) [17].

## Comorbidities:

After adjusting for confounders and comparing hypertensive patients to non-hypertensive patients, Gao and colleagues showed that hypertensive patients had a significantly higher risk of mortality from COVID-19. [18] However, they did not emphasise diabetes as a risk factor. Reviews on hypertension mentioned it as a major factor for clinical outcomes in patients with COVID-19. [19] In our study, hypertension and diabetes were marked as a major independent and combined risk factor for worst outcomes associated with COVID-19. Findings of our study also demonstrate that hypertension alone contributes to a small degree of risk for developing severe infections; but not leading to death nor development of acute respiratory failure. These findings were strong enough to withstand various sensitivity analyses and adjustments for other concomitant conditions. It's worth noting that hypertension's risk is amplified by its confounding impact on Diabetes Mellitus-Type 2. Furthermore, neither hypertension nor T2DM enhanced the risk of mortality once ARDS/respiratory failure was established. Significantly, hyperglycaemia during hospitalisation was the key driver of increased risk of all outcomes, more so than a history of T2DM, but elevated blood pressure was less contributory. Other comorbidities that were identified as risk factors for negative outcomes included advanced age, male sex, and history of other cardiovascular diseases, chronic kidney disease, and malignancies. Chronic lung illness was found to be a risk factor for severe infection development but not for mortality, ARDS, or respiratory failure.

Meta-analysis that included outcomes from 16 publications, identified hypertension, chronic kidney failure, type-2 diabetes, cardiovascular disease and obstructive pulmonary disease as significant factors associated with the development of serious illness, where patient might require ICU admission and mechanical ventilators. [20] However T2DM has a significant importance for the worst outcome like death. Since this was not a patient based meta-analysis, the study lacks to prove role of independent and dependent contribution of hypertension and T2DM. [20] Based on univariate analysis; a meta-analysis conducted by Tain et al.. reported that T2DM alone as a risk factor of mortality due to COVID-19. However, this study did not mention the contribution of hypertension alone in severity of disease. [11] According to the present study, it was analysed that; type 2 diabetes mellitus alone is responsible for more serious illness than hypertension and/ both the comorbidities together and any other conditions. More specific studies are required to analyse the severe outcome of disease associated with comorbidities.

## Haematological findings:

The outcome of the current study suggests haematological abnormalities especially anaemia having a significant association with COVID-19 and mortality. It has been documented those respiratory diseases combined with anaemia have significant effect on outcomes and increases mortality [21]. In community acquired pneumonia, anaemia is predominant amongst the patients with nearly 7 to 12% [22]. In the study by Zhou et al. [3] frequency of anaemia was recorded around 15% of 199 patients. Similarly, in cohort study of 267 subjects with COVID-19, 16% of patients had anaemia at the time of admission, whereas increase in incidence to 53% was seen during hospitalization [22]. In our study, the prevalence of anaemia was reported 34.6%, which is much higher than reported in the study by Zhou et al.. [3]. Due to a paucity of research on anaemia in COVID-19 patients; the true prevalence of anaemia in COVID-19 patients is unknown. According to results of multivariate analysis, there is significant increase in total leucocyte counts (10.64 ± 6.18; 8.87) in patients with severe infection versus non-severe (10.54 ± 5.57) leading to death. A multi centre retrospective study in China found that leukocytosis on admission of COVID-19 patients was linked to a higher probability of mortality in the hospital [23]. Non-survivors were also substantially more likely than survivors (P0.001) to have leukocytosis, according to Zhou et al. [24].

Findings of current study indicate no significant difference in lymphocyte and platelet counts amongst the severe and non-severe cases. Yang et al. [25] showed that there is an insignificant difference in lymphocytes between severe and non-severe patients and it is said that lymphopenia is not specific for COVID patients, but also prominent in elderly. Several studies suggest lymphopenia as a major contributor amongst the fatality related to COVID-19 [26-29]. Lymphocyte count is indicated as a major prognostic tool to measure severity of infection in patients with COVID-19 [30]. Data shows that the deceased were mainly of age group (50-59 years) hence, age could not be the contributor in severity of disease. However, the extent of lymphopenia and its progression among COVID-19 patients may be determined by the patients' age and clinical condition [29].

In the majority of studies, increased neutrophil count was a prominent finding [26,28,29]. In the study conducted in Singapore on 138 hospitalised patients resulted in an increase of neutrophil count amongst the patients admitted to ICU (11.6x109/L vs 3.5x109/L) [28]. Similarly, in this study neutrophil count was marked higher in severe infection patients (7.78) than non- severe patients (7.65) respectively. Qin et al. [31] and Gong et al. [32] observed significantly higher neutrophil counts in severe than non-severe patients (P<0.001), and it was also shown in a study by Li et al. among non-survivors compared to survivors. [33] Another research on 82 dead COVID-19 patients by Zhang et al. found that 74.3 percent of them had neutrophilia on admission, which rose to 100% in the 24 hours before death. [10] The cytokine storm that characterises COVID-19 sickness could be linked to the occurrence of neutrophilia. However, because neutrophilia can be caused by bacterial co-infections and the medication used to treat the condition like corticosteroids, cautious interpretation is essential. Despite the fact that the predictive capacities of haematological parameters for COVID-19 patients vary between studies, the need to include these data for early identification of high-risk patients requiring intensive care should be mandatory.

Strength and limitations: This is amongst a very few studies done on causes of mortality amongst the patients who died due to COVID-19. Hence, this helps to provide an insight of comorbidities and clinical outcomes responsible for mortality; which indeed is the strength of this study. However, this study has limitations where in; this is a single centre study hence, generalising the findings can be challenging. Also, we have measured comorbidities associated with death due to COVID-19 and have not emphasised much on pathological findings.

## Conclusion:

In this observational study, most cases of COVID-19 deaths were males over 50 years of age with different comorbidities such as, diabetes, hypertension, cardiovascular and respiratory diseases. Comorbidities are indeed risk factors that increase chances of developing ARDS leading to worst outcomes. Haematological abnormality such as anaemia was also prevalent and was independently associated with mortality amongst the patients. Moreover, the patients with comorbidities and haematological abnormalities are at a higher risk of developing severe infection such as, ARDS and death due to COVID-19. Hence, recording of comorbidities and changing haemoglobin levels throughout hospitalisation may help as a guideline to develop risk stratification and management of patients with COVID-19 to reduce overall mortality.
